# Feasibility of Biliary Drainage Using a Novel Integrated Biliary Stent and Nasobiliary Drainage Catheter System for Acute Cholangitis

**DOI:** 10.7759/cureus.37477

**Published:** 2023-04-12

**Authors:** Koji Takahashi, Hiroshi Ohyama, Yuichi Takiguchi, Motoyasu Kan, Mayu Ouchi, Hiroki Nagashima, Izumi Ohno, Naoya Kato

**Affiliations:** 1 Gastroenterology, Chiba University, Chiba, JPN; 2 Medical Oncology, Chiba University, Chiba, JPN

**Keywords:** endoscopic retrograde cholangiopancreatography, nasobiliary drainage tube, plastic biliary stent, endoscopic drainage, acute cholangitis

## Abstract

Background

Acute cholangitis is caused by cholestasis and bacterial infection, and if exacerbated, sepsis may occur and be fatal. Biliary drainage is recommended for acute cholangitis regardless of severity, except in some cases of mild acute cholangitis, in which antibiotics are effective. A novel integrated device comprising a biliary drainage stent and a nasobiliary drainage tube, called the UMIDAS NB stent (UMIDAS Inc., Kanagawa, Japan), was developed. In this study, we evaluated the efficacy and safety of biliary drainage using the UMIDAS NB stent outside type for acute cholangitis in clinical practice.

Methods

Patients with acute cholangitis with common bile duct stones or distal biliary strictures who underwent biliary drainage with the UMIDAS NB stent outside type at our institution between January 2022 and December 2022 were examined retrospectively. The UMIDAS NB stent outside type was placed transpapillary using endoscopic retrograde cholangiopancreatography (ERCP). Patients with biliary drainage stent placement other than the UMIDAS NB stent outside type on the same ERCP session and patients with acute cholecystitis were excluded.

Results

A total of 13 patients were included in this study. The severity of cholangitis was mild in four cases, moderate in five, and severe in four. There were eight cases of common bile duct stones and five cases of pancreatic cancer. The stent diameter was 7 French scale (Fr) in five cases and 8.5 Fr in eight cases. The median procedure time was 20 minutes. Clinical success was achieved in all 13 patients (100%). No treatment-related adverse events were observed. Unintended removal of the nasobiliary drainage tube was not observed. There were no cases of biliary drainage stent dislocation with nasobiliary drainage tube removal.

Conclusions

Although the sample size was small, our study demonstrated that biliary drainage with the UMIDAS NB stent outside type was effective and safe for patients with acute cholangitis who had common bile duct stones or distal biliary strictures, regardless of the severity of cholangitis.

## Introduction

Acute cholangitis develops when bile stagnation and infection are accompanied by bile duct stenosis or obstruction. Increased intraductal pressure of the bile duct causes cholangiovenous or cholangiolymphatic reflux, which leads to systemic inflammatory response syndrome [1]. The Tokyo Guidelines 2018 recommend biliary drainage for acute cholangitis regardless of its severity, except for mild acute cholangitis, in which the administration of antibiotics and general care are effective. For methods of biliary drainage, endoscopic transpapillary biliary drainage using endoscopic retrograde cholangiopancreatography (ERCP) should be considered the first-choice procedure because of its less invasiveness and lower risk of adverse events than other biliary drainage procedures, despite there being a risk of postprocedural pancreatitis [2]. Endoscopic transpapillary biliary drainage involves endoscopic biliary drainage stenting (EBS) using a stent and endoscopic nasobiliary drainage (ENBD) using a nasal drainage tube. A novel integrated device comprising a biliary drainage stent and a nasobiliary drainage tube, called UMIDAS NB stent (UMIDAS Inc., Kanagawa, Japan), was developed. A 7 French scale (Fr) or 8.5 Fr biliary drainage stent and nasobiliary drainage tube are integrated, and these devices allow simultaneous biliary drainage stent and nasobiliary drainage tube placement with one device insertion in biliary drainage using the ERCP technique. There is no need to insert the device into the bile duct twice; this is the least invasive approach available for the management of acute cholangitis and biliary drainage [3]. Its use in the treatment of acute cholangitis allows a nasobiliary drainage tube to monitor bile drainage and irrigate bile ducts with saline after placement. After cholangitis has improved, only the biliary drainage stent remains if the nasobiliary drainage tube is removed without additional ERCP. There are two types of UMIDAS NB stents, inside type and outside type. These two types have the same nasobiliary drainage tube but different biliary stents. Stents must be placed across stones or tumors for effective drainage. The inside type stent is used when there is a pathology involving the hilar region of the bile duct. The outside type stent is used when there is a pathology involving either the hilar region or the distal region of the bile duct. An inside stent is designed such that its distal end is positioned within the bile duct. It is intended to prevent the backflow of food and digestive fluids into the bile duct. On the other hand, an outside stent was designed such that its distal end was located in the duodenal lumen and is versatile. It can be used in a variety of situations, regardless of the location of the biliary stenosis or the location of the stone. In this study, we investigated the efficacy and safety of biliary drainage using the UMIDAS NB stent outside type for acute cholangitis.

## Materials and methods

Study design

This retrospective clinical study was conducted at a single center. Patients with acute cholangitis with common bile duct stones or distal biliary strictures who underwent biliary drainage with the UMIDAS NB stent outside type at our institution between January 2022 and December 2022 were examined using medical records. Patients with another biliary drainage stent placement in addition to the UMIDAS NB stent outside type on the same ERCP session and patients with concurrent acute cholecystitis were excluded. The primary outcome of this study was the clinical success rate of the ERCP procedure using the UMIDAS NB stent outside type for acute cholangitis and the secondary endpoint was its safety.

Written informed consent for the procedure was obtained from all patients. This study was approved by the Ethics Committee of our institution, Chiba University. Consent for patient participation in this study was obtained using the opt-out methodology. This study was conducted in accordance with the principles of the Declaration of Helsinki.

Definitions

According to the Tokyo Guidelines 2018, acute cholangitis was diagnosed and its severity was determined [4]. The procedure time was measured from duodenoscope insertion to its removal. Based on the Tokyo Criteria 2014, clinical success was defined as a 50% reduction in serum bilirubin levels within 14 days, or in cases without jaundice, no jaundice was assumed after the procedure [5]. Adverse events were graded according to the Tokyo Criteria 2014. Regarding operators, endoscopists with more than seven years of ERCP experience were defined as experts, and others were defined as trainees. Regarding endoscopic sphincterotomy (EST), it was categorized as performed during the procedure, previously performed, and not performed.

Techniques

**Figure 1:**
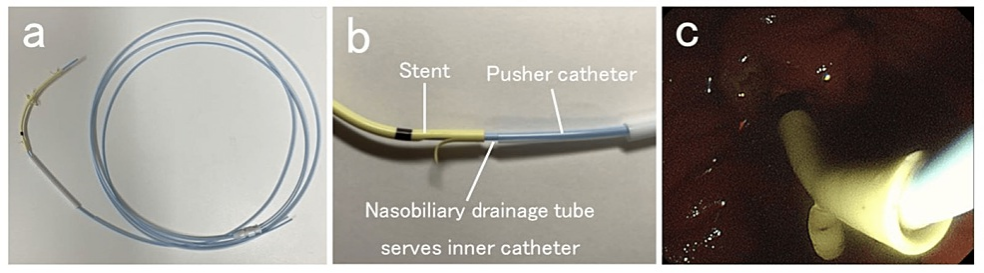
Structure of the UMIDAS NB stent outside type (a) (b) The UMIDAS NB stent outside type (UMIDAS Inc., Kanagawa, Japan) consists of a stent, a nasobiliary drainage tube (also serving as the inner catheter tube), and a pusher tube. (c) Using endoscopic retrograde cholangiopancreatography, insert the UMIDAS NB stent outside type transpapillary into the bile duct. After the pusher tube is pulled out, remove the endoscope, leaving the stent and nasobiliary drainage tube.

**Figure 2:**
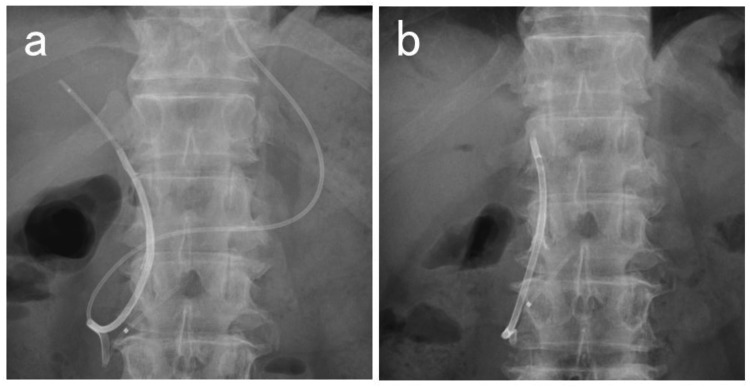
Removal of nasobiliary drainage tube in the UMIDAS NB stent outside type (a) The UMIDAS NB stent outside type (UMIDAS Inc., Kanagawa, Japan) was placed. (b) The nasobiliary drainage tube of the UMIDAS NB stent outside type was removed, leaving only the stent.

The UMIDAS NB stent outside type was placed using the ERCP technique (Figure 1). Intravenous antibiotics were administered before ERCP. Carbon dioxide insufflation was used during the procedure unless contraindicated. All patients underwent conscious sedation with the administration of a combination of midazolam and pethidine hydrochloride or fentanyl and propofol. An oblique-viewing duodenoscope (TJF-260V, and TJF-Q290V; Olympus, Tokyo, Japan) was inserted orally to reach the duodenal papilla, and an ERCP catheter (PR-104Q-1; Olympus, Tokyo, Japan. MTW ERCP catheter; ABIS, Hyogo, Japan) was inserted into the bile duct. After imaging the bile duct with a contrast medium, a guidewire (VisiGlide2; Olympus, Tokyo, Japan. M-Through; ASAHI INTECC, Aichi, Japan. EndoSelector; Boston Scientific, Marlborough, MA, USA) was placed in the bile duct followed by the UMIDAS NB stent outside type insertion without stone removal or stricture dilation. The UMIDAS NB stent outside type was then placed by removing the endoscope while pushing the device, and the end of the tube was made to exit the mouth. Subsequently, a silicone rubber catheter was inserted through the nose and pull the tip out of the mouth. The nasobiliary drainage tube and catheter were connected, the catheter was pulled, and the tube was pulled out through the nose and left in place. After placement of the UMIDAS NB stent outside type, the bile duct was washed with 20-40 mL of saline using a nasobiliary drainage tube. After an improvement of acute cholangitis, the nasobiliary drainage tube was removed by pulling it at the bedside, leaving only the biliary drainage stent (Figure 2).

Statistical analysis

Data are presented as median with range or number with percentage. All statistical analyses were performed using the Bell Curve for Excel (Social Survey Research Information Co., Ltd., Tokyo, Japan).

## Results

A total of 13 eligible patients were included in this study. The eligible patients’ ages ranged from 58 to 87 years, with an average age of 77 years. Table 1 summarizes the patient characteristics and clinical outcomes. Table 2 presents the results of the data analysis. The severity of cholangitis was mild in four cases, moderate in five, and severe in four. Trainees accounted for 92.3% of operators. There were eight cases of common bile duct stones and five cases of pancreatic cancer. A native papilla was observed in seven cases, and among them, EST was performed in four cases in the same session as the UMIDAS NB stent outside type placement. Among the three patients who did not undergo EST, one patient was taking two antiplatelet drugs and two patients were taking warfarin. The biliary drainage stent diameter of the UMIDAS NB stent outside type was 7 Fr in five cases and 8.5 Fr in eight cases. The median procedure time was 20 minutes. Clinical success was achieved in all 13 patients (100%). No treatment-related adverse events were observed. Regarding the nasobiliary drainage tube, two patients were transferred to another hospital with a nasobiliary drainage tube in place. Among the 11 cases in which the nasobiliary drainage tube was removed at our hospital, the median duration of the nasobiliary drainage tube placement was three days. Unintended removal of the nasobiliary drainage tube was not observed. There were no cases of biliary drainage stent dislocation with nasobiliary drainage tube removal.

**Table 1:** A summary of the patient's characteristics and clinical outcomes Fr, French scale

Case	Age, year	Sex	Severity of cholangitis	Operator	Cause of cholangitis	Native papilla	Endoscopic sphincterotomy	Diameter of the stent, Fr	Procedure time, minutes	Adverse events	Clinical success	Nasobiliary drainage tube retention period, days
1	66	Male	Moderate	Trainee	Common bile duct stone	No	Previously performed	7	26	No	Yes	2
2	58	Female	Severe	Trainee	Common bile duct stone	Yes	Not performed	7	20	No	Yes	3
3	83	Male	Moderate	Trainee	Common bile duct stone	No	Previously performed	8.5	50	No	Yes	2
4	87	Male	Moderate	Trainee	Pancreatic cancer	No	Previously performed	8.5	20	No	Yes	4
5	79	Male	Severe	Trainee	Common bile duct stone	Yes	Performed during the procedure	8.5	24	No	Yes	6
6	63	Male	Severe	Trainee	Common bile duct stone	Yes	Performed during the procedure	7	14	No	Yes	Transferred to another hospital before removal
7	68	Female	Mild	Trainee	Pancreatic cancer	No	Previously performed	8.5	17	No	Yes	4
8	60	Female	Mild	Trainee	Common bile duct stone	Yes	Performed during the procedure	8.5	23	No	Yes	Transferred to another hospital before removal
9	77	Male	Moderate	Trainee	Common bile duct stone	Yes	Not performed	7	28	No	Yes	1
10	80	Male	Mild	Trainee	Pancreatic cancer	No	Previously performed	8.5	16	No	Yes	2
11	74	Female	Mild	Trainee	Common bile duct stone	Yes	Not performed	7	23	No	Yes	3
12	83	Female	Severe	Expert	Pancreatic cancer	No	Previously performed	8.5	15	No	Yes	34
13	81	Male	Moderate	Trainee	Pancreatic cancer	Yes	Performed during the procedure	8.5	19	No	Yes	8

**Table 2:** The results of analyzing the data Fr, French scale

Valuable	n=13
Age, year, median (range)	77 (58-87)
Sex, male, number (%)	8 (61.5)
Severity, number (%)	
Mild	4 (30.8)
Moderate	5 (38.5)
Severe	4 (30.8)
Operator, number (%)	
Expert	1 (7.7)
Trainee	12 (92.3)
Cause of cholangitis, number (%)	
Common bile duct stone	8 (61.5)
Pancreatic cancer	5 (38.5)
Native papilla, number (%)	7 (53.8)
Endoscopic sphincterotomy, number (%)	
Performed during the procedure	4 (30.8)
Previously performed	6 (46.2)
Not performed	3 (23.1)
Diameter of the stent, number (%)	
7Fr	5 (38.5)
8.5Fr	8 (61.5)
Procedure time, minutes, median (range)	20 (14-50)
Clinical success, number (%)	13 (100)
Adverse events, number (%)	0
Nasobiliary drainage tube retention period, days, median (range)	3 (1-34)

## Discussion

In this study, the UMIDAS NB stent outside type was effective in all cases regardless of the severity and cause of acute cholangitis. No adverse events were observed. For acute cholangitis, reliable drainage with a nasobiliary drainage tube was performed for a while after ERCP, and only the stent remaining after the nasobiliary drainage tube was removed without additional ERCP.

The UMIDAS NB stent has both EBS and ENBD functions. EBS is an internal drainage technique, and its advantages include the absence of tube discomfort, water loss, or electrolyte loss. However, early occlusion may occur as a disadvantage in cases where bile is highly viscous. On the other hand, ENBD is an external drainage technique, and its advantages include the ability to monitor bile quantity and color and wash the bile ducts with saline. It is particularly useful if bile is purulent and viscous. However, the disadvantages include unintended removal and tube discomfort. Several randomized controlled trials have reported the use of ENBD and EBS as drainage methods for acute cholangitis. In 2002, Lee reported that ENBD and EBS were equally effective in acute cholangitis caused by bile duct stones; however, ENBD had tube dislodgement in 10% and tube kink in 2.5% of patients, and there was a significantly lower mean patient discomfort score on day 1 after the procedure in the stent group [6]. In 2005, Sharma reported that ENBD and EBS were equally effective in treating severe cholangitis, with no unintended withdrawals or kinks in the ENBD group [7]. In 2013, Zhang reported that the clinical manifestations of ENBD and EBS were similar; however, there was a significantly lower patient discomfort score in the EBS group, and the incidence rate of occlusion in EBS was significantly higher than that in ENBD [8]. In the updated Tokyo Guidelines 2018, a meta-analysis that included three randomized controlled trials showed no statistically significant difference in technical success, clinical success, adverse events, or reintervention rates between ENBD and EBS, suggesting that either ENBD or EBS may be considered for biliary drainage according to the patient’s background and preference [2].

Using the UMIDAS NB stent outside type, reliable drainage using a nasobiliary drainage tube can be expected after ERCP by monitoring the bile and having the ability to wash the bile ducts with saline. Even if the nasobiliary drainage tube is unintentionally removed, the biliary drainage stent remains in place. This method is particularly useful in cases of severe cholangitis requiring reliable drainage. Compared to placing a stent and nasobiliary drainage tube separately, only one device is required; therefore, the procedure is simple and cost-effective for placing the UMIDAS NB stent outside type.

To date, most reports on the UMIDAS NB stent are case reports, and although there are almost no reports of comprehensive usage experience, its usefulness in various situations has been reported. Iwano reported that by using multiple UMIDAS NB stents inside types for cholangitis due to perihilar biliary obstruction, by removing the nasobiliary drainage tube after improvement of cholangitis, multiple stents can be left without additional ERCP [9]. Mandai reported that the use of the UMIDAS NB stent inside type in transpapillary drainage for acute cholecystitis could prevent cholecystitis recurrence by leaving the gallbladder drainage stent without performing ERCP after cholecystitis has improved [10]. Regarding adverse events, there have been some reports of the UMIDAS NB stent outside type. Yamamoto made two reports on the adverse events of the UMIDAS NB stent outside type. In the first report, when the UMIDAS NB stent outside type was placed after endoscopic papillectomy for ampullary adenoma, the stent subsequently penetrated the duodenal wall after migrating into the bile duct [11]. In the second report, the UMIDAS NB stent outside type was placed after endoscopic papillectomy for ampullary adenoma. The stent then blocks the cystic duct, causing acute cholecystitis in two cases [12].

In this study, unintended removal of the nasobiliary drainage tube was not observed, but unintended removal of the nasobiliary drainage tube was one of the serious concerns. Using the UMIDAS NB stent outside type, the biliary drainage stent was retained when the nasobiliary drainage tube was removed unintentionally. It can eliminate the burden of performing ERCP again on the patient, and reduce adverse events by reducing the frequency of ERCP. The UMIDAS NB stent outside type needs a device setting, and staff needs to get used to it. The UMIDAS NB stent outside type has EBS and ENBD functions and can be placed at a lower cost than placing both EBS and ENBD as separate products. The UMIDAS NB stent outside type can be the first-choice device for acute cholangitis. There have been few comprehensive reports on the use of the UMIDAS NB stent outside type. Although this study included a small number of cases, it is a valuable report showing the usefulness of the UMIDAS NB stent outside type for acute cholangitis.

The limitations of this study include its retrospective design, lack of a control group, and small sample size.

## Conclusions

In this study, we reported the initial outcomes of the UMIDAS NB stent outside type for acute cholangitis. Although the sample size was small, our study demonstrated that biliary drainage with the UMIDAS NB stent outside type was effective and safe for patients with acute cholangitis who had common bile duct stones or distal biliary strictures, regardless of the severity of cholangitis.
